# Genotypes of *Giardia duodenalis* in Household Dogs and Cats from Poland

**DOI:** 10.1007/s11686-020-00292-1

**Published:** 2020-10-11

**Authors:** Agnieszka Piekara-Stępińska, Jolanta Piekarska, Michał Gorczykowski, Jacek Bania

**Affiliations:** 1 Division of Parasitology, Department of Internal Medicine and Clinic of Diseases of Horses, Dogs and Cats, Wrocław University of Environmental and Life Sciences, CK Norwida 31, 50-375 Wrocław, Poland; 2 Department of Food Hygiene and Consumer Health Protection, Wrocław University of Environmental and Life Sciences, CK Norwida 31, 50-375 Wrocław, Poland

**Keywords:** Nested PCR, β-Giardin, Protozoa, Zoonosis

## Abstract

**Background:**

*Giardia duodenalis* is a widespread protozoan parasite affecting humans and many species of animals, including dogs and cats. Due to its zoonotic potential, it is important to know the frequency of this parasite in companion animals. The aim of this study was to determine current epidemiological status of *G. duodenalis* in household dogs and cats.

**Methods:**

In this study, 293 fecal samples from pet dogs and cats were collected from January 2017 to July 2019 and tested for *G. duodenalis* by PCR (using β-giardin gene). The animals were divided into groups depending on their age, breed and fecal consistency.

**Results:**

The examination allowed for detection of *G. duodenalis* in 6.0% of canine and 3.9% of feline fecal samples. The highest frequency was revealed in young (under one-year old) dogs. Sequencing confirmed the presence of assemblages C and D in dogs and A and F in cats.

**Conclusion:**

The study showed current frequency of *G. duodenalis* in dogs and cats and also revealed the occurrence of host-specific assemblages as well as zoonotic assemblage A.

## Introduction

Dogs and cats are intimate companion animals of humans. According to data from 2019, there are over seven million dogs and over six million cats in Poland [40]. Despite numerous advantages of having a pet, close contact between humans and dogs or cats can result in zoonotic diseases. Important factors causing zoonotic diseases are parasites, such as *Giardia duodenalis, Cryptosporidium *spp.,* Echinococcus *spp.,* Dipylidium caninum* or *Toxocara* spp.

*Giardia duodenalis* (also known as *Giardia intestinalis* or *Giardia lamblia*) is a unicellular protozoan parasite affecting humans and many animal species. There are two morphological stages of *G. duodenalis*: a trophozoite and a cyst (infective stage). The parasite can colonize the upper small intestine but it was also found in the lower small intestine, stomach, colon and biliary tract [3, 7]. *G. duodenalis* transmission occurs via fecal–oral route (from contaminated water or food and directly from infected individuals) [5]. Giardiasis in dogs and cats can include diarrhea and weight loss or the disease can be asymptomatic.

In recent years, the infection rate of *G. duodenalis* in Europe ranged from 0.8% (Switzerland) to 42% (Germany) and from 5.9% (Spain) to 20.5% (Greece) in dogs and cats, respectively [11, 21, 31, 42].

Studies conducted in Poland between 2006 and 2017 revealed the frequency of *G. duodenalis* to range from 2 to 36% in dogs and from 3.2 to 15.1% in cats, depending on the examined population, geographical origin of the animal, and diagnostic methods (Table 1). The assemblages detected so far in Poland are B, C, D in dogs and A, B, D, F in cats. However, due to small areas covered by the previous studies, their outcomes did not reflect epidemiological situation for the entire area of Poland [4, 19, 29, 34, 43].

**Table 1:** *Giardia duodenalis* in dogs and cats in Poland, 2006–2017

Area of Poland	Populations	No. of examined samples	No. of positive samples	Infection rate (%)	Method	Assemblages (if examined)	References
*Dogs*							
Warsaw	Owned	350	18	5.14	Microscopy	A-I, C, D	[43]
			–	9.14	PCR		
Unknown	Sled	64	–	35.9	IFA		[4]
West-central region of Poland	Sheltered	88	2	2.3	Microscopy^a^	C, D	[34]
	Owned	60	1	1.6			
Wroclaw	Owned	128	27	21.1	PCR (bg)	B, C, D	[29]
*Cats*							
Warsaw	Household	160	6	3.75	Microscopy^a^	A, B, D	[19]
Wroclaw	Owned	33	5	15.1	PCR (bg)	F, A	[29]
Unknown	Pet	31	1	−	PCR	F	[22]
	stray	33	2	−		F	

^a^DNA was isolated from microscopy-positive samples only

Since 2004 giardiasis has been considered by WHO a neglected disease [32]. Human giardiasis can be asymptomatic or can cause persistent diarrhea or malabsorption associated with body weight loss [13].

*G. duodenalis* includes eight morphologically indistinguishable assemblages (A–H). The assemblages A and B are further divided into sub-genotypes AI, AII, AIII, BIII and BIV. Typical genotypes in dogs are C and D, but A and B can also be found, and exceptionally even E and F ones [6, 9, 12, 14, 20]. Genotype F is common in cats, which can be infected also by genotype A, E and rarely C [6, 21, 24]. Humans are almost exclusively infected by assemblages A and B but genotypes C, D, E and F were also found in rare cases [1, 6, 14, 30, 37].

Microscopic studies using fecal flotation enable detection of the cysts [35]. Other diagnostic methods involve detection of coproantigen, usually by ELISA. PCR techniques, based on the amplification of gene fragments encoding SSU rRNA, glutamate dehydrogenase (*gdh*), triosephosphate isomerase (*tpi*) or β-giardin (*bg*), allow for detection of *Giardia* DNA and also for genotyping [15, 26]. Although the multilocus genotyping is considered the most useful, genotyping based on single locus with high sequence heterogeneity (such as *bg* or *tpi*) is commonly accepted, especially where the diagnosis is extended to the sequencing of the obtained PCR products [23, 36]. One of the most commonly used markers is β-giardin, which allows for a successful detection of the parasite by PCR and also enables genotyping and subgenotyping of assemblage A [5, 23]. β-giardin allows also for identification of mixed invasions, especially in the case of two-way analysis of the obtained sequences [31]. Moreover, one of the most sensitive and specific methods for detection of *Giardia* spp. is immunofluorescence and it is considered a reference standard assay for the detection of this parasite in dogs and cats feces [16, 38].

Due to the zoonotic potential of *G. duodenalis*, it is particularly important to determine its current infection rate in domestic animals. The overall prevalence and frequency of *G. duodenalis* genotypes in dogs and cats can indicate the potential risk of invasion in humans. The aim of this study was to run a molecular detection of *G. duodenalis* in fecal samples and to assess its overall frequency broken into frequency of each genotype in household dogs and cats from Poland.

## Methods

### Study area and sample collection

**Fig. 1:**
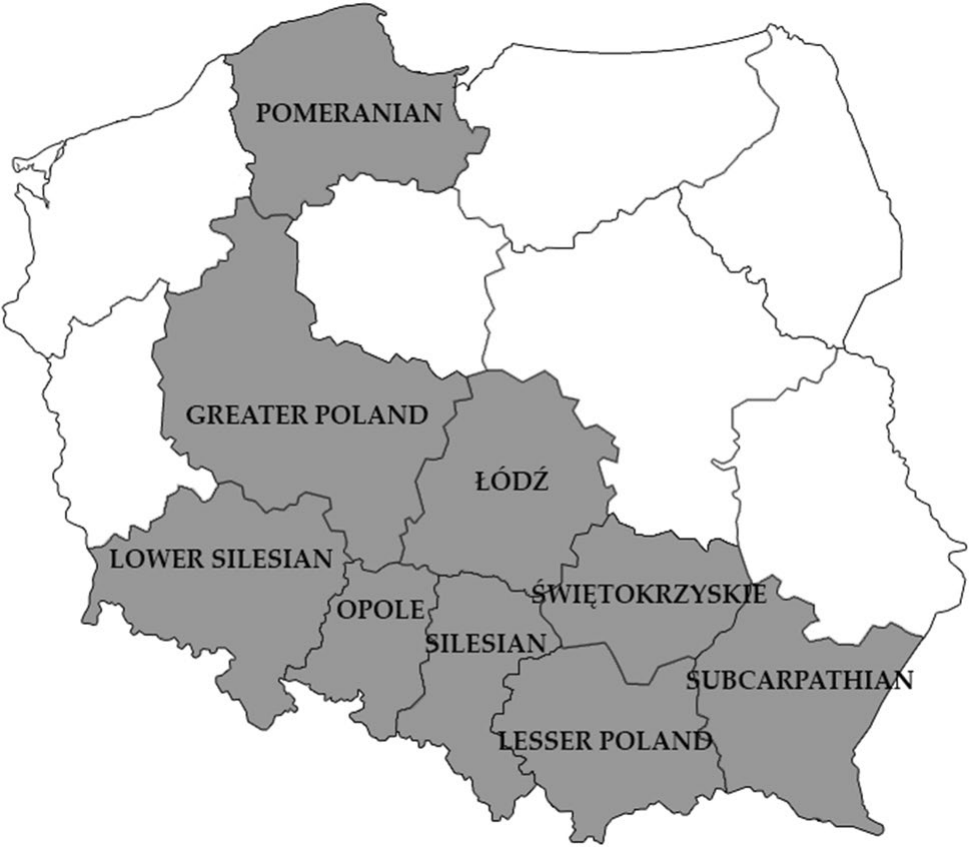
Map of Poland. Dark grey areas (voivodeships) represent the origin of the tested canine fecal samples. Images used are in the public domain and were downloaded from: https://pl.wikipedia.org/wiki/Plik:Polska_kontur_bialy.png

A total of 293 fresh fecal samples were obtained between January 2017 and July 2019 from individual, randomly chosen household dogs (217 samples) and cats (76 samples) living in different regions of Poland. The area of Polish territory is over 312,000 square kilometers divided into 16 provinces. The examined samples came from nine provinces (Pomerania, Greater Poland, Lower Silesia, Opolskie Voivodship, Silesia, Lodzkie Voivodship, Holy Cross, Lesser Poland, Subcarpathian), which account for nearly 153,000 square kilometers (about 50% of the country area) (Fig. 1). The samples, collected by pet owners, were placed individually into disposable plastic bags. The age of the animals ranged from nine weeks to eleven years. They were grouped based on the age (under one-year old, over one-year old), breed and feces consistency (formed, unformed). About 1 g of each sample was frozen at -80˚ C for further analysis.

### DNA extraction

DNA was isolated from 100 mg of each fecal sample using Genomic Mini AX Stool (A&A Biotechnology, Poland) as per the manufacturer’s instruction. The DNA samples were stored at – 80 °C until further use.

### PCR amplification

To identify *G. duodenalis* in the stool samples, fragments covering β-giardin gene were amplified by nested PCR. The amplification of a 763 bp region was carried out using a forward primer G7 (5′AAGCCCGACGACCTCACCCGCAGTGC3′) and a reverse primer G759 (5′GAGGCCGCCCTGGATCTTCGAGACGAC3′). For secondary PCR, 587 bp fragment was amplified using 1 µl of the first PCR product. The secondary PCR was carried out using nested forward 511 (5′ GAACGAACGAGATCGAGGTCCG’3) and nested reverse 511 (5′ CTCGACGAGCTTCGTGTT 3′). The mixture composition and PCR conditions were described by Lalle et al. (2005) [23]. PCR mix consisted of a buffer containing 1.5 mM MgCl_2_, 200 mM of each dNTP (dNTP mix 10, A&A Biotechnology, Gdynia, Poland) 10 pmol of each primer, 2.5 units of RUN DNA polymerase (A&A Biotechnology, Gdynia, Poland) and 3 µl (1 µl in secondary PCR) of purified DNA in a final volume of 25 µl. PCR was performed using a thermocycler BioRad T100™ Thermal Cycler. Primary PCR conditions were as follows: 95 °C for 5 min for 1 cycle, 95 °C for 30 s, 50 °C for 30 s and 72 °C for 1 min for 40 cycles followed by 72 °C for 7 min. Secondary PCR conditions were: 96 °C for 5 min for 1 cycle, 96 °C for 45 s, 55 °C for 30 s and 72 °C for 45 s for 35 cycles followed by 72 °C for 7 min. The secondary PCR products were examined electrophoretically in 2% agarose gels and visualized after staining with Midori Green Advance DNA Stain (Genetics, Germany).

### DNA sequencing and data analysis

PCR products purification and sequencing was performed by Genomed (Poland) in both directions. The resulting chromatograms were visually assessed to exclude the presence of double peaks. The obtained sequences were compared by a blast search (https://blast.ncbi.nlm.nih.gov/Blast.cgi) with sequences deposited in GenBank. Phylogenetic analysis was performed using neighbor-joining method with MEGA 4 free software. Evaluation of the reliability of the clusters was confirmed using Bootstrap values (1000 replicates). Reference sequences used in the phylogenetic tree were: AB508814.1 for assemblage A; MN270296.1, KX757753.1 for assemblage C; JN416550.1, JN416548.1, JN416559.1 and LC316659.1 for assemblage D and LC341557.1 for assemblage F.

### Statistical analysis

The frequency of *G. duodenalis* infections presented in the tables shows the percentage of positive samples in the studied population. We also provided confidence intervals (CI) at the level of 95% (*p* = 0.05), calculated according to the Wilson method. The chi-square test (*χ*^2^) with Yates correction implemented in STATISTICA ver. 12.0 software package was used to compare the differences in *Giardia* infection rates among the investigated groups. Differences were considered significant at *p* ≤ 0.05.

## Results

**Fig. 2:**
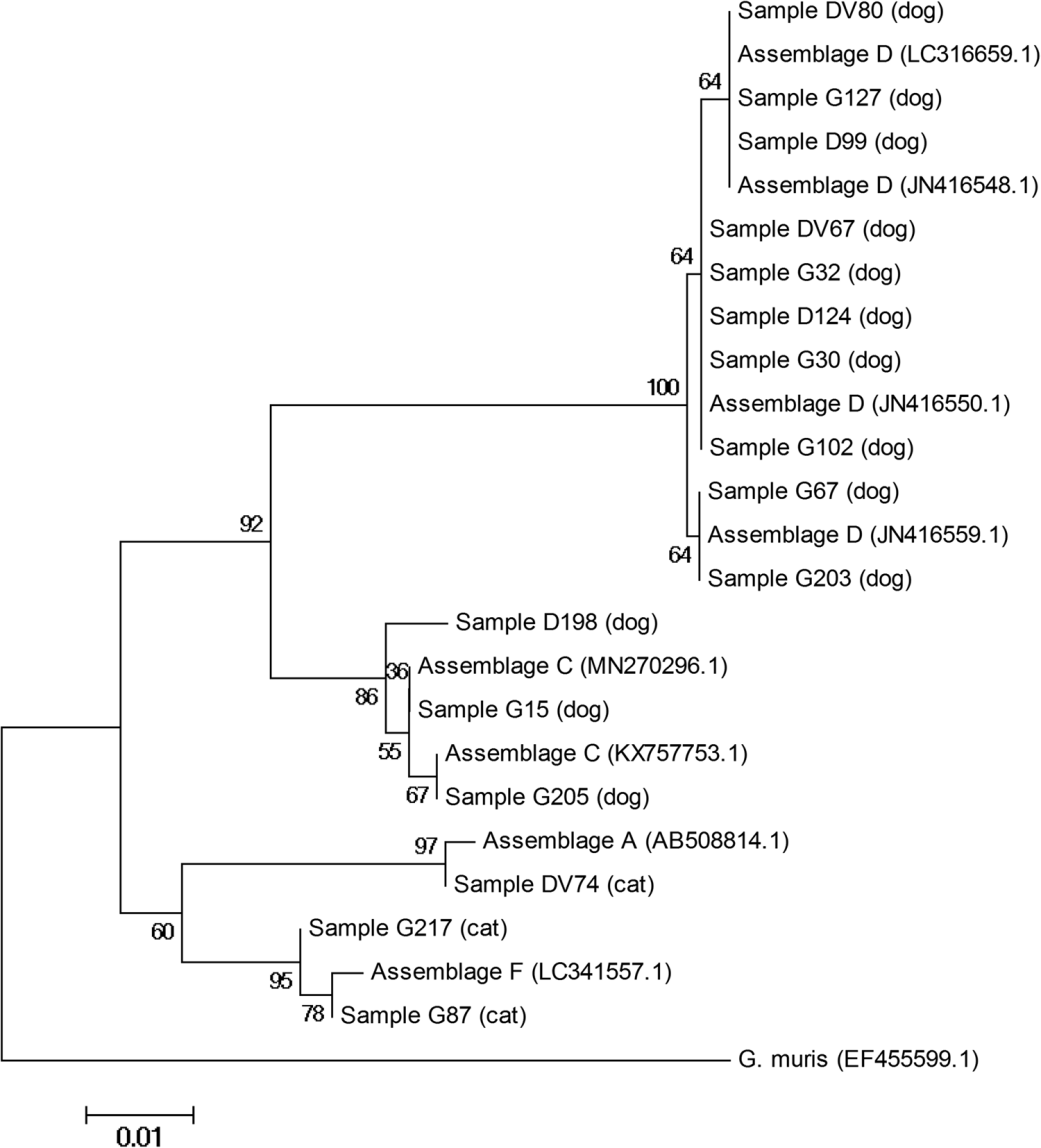
Phylogenetic relationship between *Giardia duodenalis* isolates collected from dogs and cats, based on sequences of β-giardin locus, determined by the neighbor-joining method, using Kimura-2 parameter model. Each branch shows bootstrap values. Reference sequences with their accession numbers in GenBank are provided. Sequence of *Giardia muris* was used as outgroup

The presence of *G. duodenalis* DNA was detected in 13/217 (6.0%) canine and in 3/76 (3.9%) feline fecal samples. Higher frequency of the infection was observed in dogs under 1 year old (13/107, 12.2%) (*p* < 0.05). Among the dog breeds, *Giardia* infections were most frequently observed in French bulldogs (4 out of 16; 25%), and the infection rate was significantly higher than in other breeds (*p* < 0.05). There were no statistically significant differences connected with age and breed in cats or fecal consistency in both species (Table 2). All 16 PCR-positive samples were successfully sequenced. In dogs, 10 isolates were the closest to assemblage D (77%) and 3 to assemblage C (23%). In cats, two were the closest to assemblage F (67%), and one to assemblage A (33%). Detailed data are included in Table 3. The phylogenetic relationship of *Giardia* isolates and reference sequences for A, C, D and F assemblages are showed in Fig. 2.

**Table 2:** Occurrence of *Giardia duodenalis* in dogs and cats in relation to clinical symptoms

Animal species (n)	Feces condition (n)	No. of positive animals	Infection frequency (CI^a^)	Sample symbol	Animal age (months)	Animal breed	Giardia genotype
Dog (217)	Formed (64)	3	4.7 (1.6–12.9)	DV80	3	West Highland White Terrier	D
				G32	2	American Staffordshire Terrier	D
				D99	9	Greater Swiss Mountain Dog	D
	Unformed (153)	10	6.5 (3.6–11.6)	G67	2	French bulldog	D
				D124	5	French bulldog	D
				DV67	9	Siberian Husky	D
				G30	6	Dachshund	D
				G102	2	German Shepherd	D
				G127	3	Siberian Husky	D
				G203	2	French bulldog	D
				G198	3	French bulldog	C
				G205	2	German Shepherd	C
				G15	2	Berger Blanc Suisse	C
Cat (76)	Formed (34)	2	5,9% (0.7–19.7)	DV74	4	Exotic Shorthair	A
				G217	6	Mixed breed	F
	Unformed (42)	1	2.4% (0.1–12.6)	G87	13	Mixed breed	F

^a^CI = 95% confidence interval according to the modified (adjusted) Wald method

**Table 3:** Comparison of *Giardia duodenalis* isolates (genotyping β-giardin gene) in dogs and cats in Poland

Host	Assemblage	Sample	Reference sequence	Stretch	SNPs
Dog	D	G67	JN416559.1	45–471	None
Dog	D	G203	JN416559.1	45–471	None
Dog	D	D124	JN416559.1	45–471	T115C
Dog	D	G32	JN416559.1	45–471	T115C
Dog	D	DV67	JN416559.1	45–471	T115C
Dog	D	DV80	JN416559.1	45–471	A67T, G109A, T115C
Dog	D	G30	JN416559.1	45–471	T115C
Dog	D	G102	JN416559.1	45–471	T115C
Dog	D	G127	JN416559.1	45–471	G109A, T115C
Dog	D	D99	JN416559.1	45–471	G109A, T115C
Dog	C	G205	KX757753.1	64–410	None
Dog	C	D198	KX757753.1	64–410	G175C, T207C
Dog	C	G15	KX757753.1	64–410	T207C
Cat	A	DV74	AB508814.1	97–559	T419C
Cat	F	G217	LC341557.1	20–452	T100C, T268C
Cat	F	G87	LC341557.1	20–452	T49G, T100C

## Discussion

The occurrence of *G. duodenalis* in dogs and cats depends mainly on the examined area but also on the diagnostic methods or examined groups (different living condition or age). In this study, the frequency of *G. duodenalis* found in fecal samples of dogs from different regions of Poland was 6.0% and this result fell within the lower limit of previous results from Poland [4, 29, 34, 43]. Assessment of this frequency was based on amplification of *bg* locus, a highly sensitive and widely recommended marker [9, 23]. However, some authors reported that DNA amplification can be difficult due to PCR inhibitors in feces [38]. Our study showed lower percentage of positive samples (6%) than some other recent publications on household dogs in Europe involving molecular methods (29% in Spain, 12.9% in Greece, 42% in Germany) [11, 21, 31]. High frequency of *Giardia* noted in Germany can be connected with lower number of examined samples and this result cannot be representative for the entire country. In our study, significantly higher frequency of the parasite was noted in young dogs, under one-year old (14%). The age of the dogs seems to be an important risk factor for *G. duodenalis,* as previously described. A recent study conducted by Pan et al. (2018), based on amplification of *bg* and *tpi* genes, showed significantly higher detection rate of *Giardia* in young (16.1%) than in adult dogs (7.6%) [28]. Shin et al. (2015) also used *bg* gene as a molecular marker and confirmed significantly higher prevalence in young, sheltered dogs [33]. We found no cases of *G. duodenalis* in the dogs over one-year old, however, many studies detected the presence of this parasite also in adult dogs [21, 28]. Among the examined breeds, French bulldogs were the most often affected by the parasite. This breed is commonly considered to be predisposed to many diseases, especially connected with respiratory and reproductive systems [27]. There are many opinions about pathogenicity of giardiasis in both humans and animals. Mochizuky et al. (2001) noticed almost equal frequency of *G. duodenalis* in symptomatic and asymptomatic dogs [25]. We found no statistically significant differences connected with fecal consistency in the examined groups, however, some authors showed higher prevalence of the parasite in diarrheic dogs. For example, higher prevalence of *G. duodenalis* in household dogs with loose consistency of feces (all dogs older than 6 months) was reported by Ulterwijk et al. (2019), but in other groups of dogs (sheltered or hunting), significant differences were not observed [17, 33, 41].

In the current study, *G. duodenalis* was found in 3.9% of feline fecal samples. These results were similar to those obtained in household cats from Spain (5.9%), but lower than in Germany (14%) and Greece (15.6%) [11, 21, 31]. We found no statistically significant differences regarding age, breed or fecal condition in cats.

In dogs, we confirmed only host-specific genotypes D (77% of positive samples) and C (23% of positive samples). This differed significantly from the results previously described for western Poland, where mostly genotype C, and just a few cases of genotypes D and B were detected [29]. The occurrence of only genotype D in Poland was reported for central and western part of the country, although there were only two specimens sequenced (34). In other European countries, dog-specific genotypes were found with the highest frequency, but in some areas genotypes A and B, and in rare cases, E and F were also detected [2, 21, 31, 35]. In northern Spain, assemblages A and B were found even more often than dog-specific genotypes [17]. In cats, we found feline-specific assemblage F (67%), but also assemblage A (33%). The previous study from Poland also showed occurrence of these genotypes [29]. In some countries neighboring Poland (Germany, Czech Republic or Slovak Republic), genotype F was the prevailing or the only one [22, 35]. Contrary to that, examination of feline fecal samples from Greece revealed mostly genotype A and rare cases of assemblages F, but also B and C [21]. The role of companion animals as a source of human giardiasis was widely discussed. Some papers showed that dogs and cats do not seem to play an important role as reservoirs of zoonotic genotypes and transmission from these animals to humans is rare [11, 31]. However, other studies proved that also genotypes A or B can be common in pet animals, what suggests potential zoonotic risk and possible consequences for human health [10, 18]. Also, Traub et al. (2004) showed strong association between giardiasis in humans and dogs from the same community [39]. Zoonotic risk seems to be different in various areas and should be assessed in each region.

In summary, fecal samples of dogs and cats from different regions of Poland were collected and PCR examination based on β-giardin locus amplification was conducted. This examination confirmed the occurrence of *G. duodenalis* genotypes specific for dogs or cats and also the occurrence of zoonotic genotype A in cats. The study confirmed that giardiasis in dogs is strongly connected with their age and is often diagnosed in dogs under one-year old. Moreover, *G. duodenalis* was found more often in French bulldogs than in the dogs of other breeds, which according to our knowledge is the first communication of such a result.
